# Deciphering Enzymatic and Phenolic Defence Signatures in Beans in Response to Halo Blight and Common Bacterial Blight Pathogens

**DOI:** 10.3390/life16101644

**Published:** 2026-09-30

**Authors:** Badel Uysal Sahin, Kubilay Kurtulus Bastas

**Affiliations:** 1 Mersin Agricultural Quarantine Directorate, 33130 Mersin, Turkey; badeluysal@hotmail.com; 2 Department of Plant Protection, Faculty of Agriculture, Selcuk University, 42130 Konya, Turkey

**Keywords:** bean, enzyme, bacteria, resistance, defence, phenolics

## Abstract

*Pseudomonas savastanoi* pv. *phaseolicola* (Psp) and *Xanthomonas axonopodis* pv. *phaseoli* (Xap) are among the most economically significant bacterial pathogens of common bean (*Phaseolus vulgaris* L.), responsible for severe yield reductions worldwide. In this study, we investigated the biochemical and physiological basis of differential defence responses in the susceptible cultivar Aras 98 and the resistant genotype 36K following infection with Psp and Xap. Four-week-old plants of the resistant genotype 36K and susceptible cv. Aras 98 were separately inoculated with Psp (510-p and 522-p) or Xap (120-x and 145-x) at 10^8^ CFU mL^−1^, with mock-inoculated plants serving as controls. Three independent biological replicates were used at each sampling time point, and leaf tissues were collected at 0, 12, 24, 36, and 72 h post-inoculation. The activities of Superoxide Dismutase (SOD), Polyphenol Oxidase (PPO) and Phenylalanine Ammonia-Lyase (PAL), as well as proline and total phenolic compounds, were subsequently evaluated. In addition, leaf surfaces collected 24 h after inoculation were examined using SEM to visualize bacterial colonization and biofilm formation. Results revealed stronger and time-dependent defence responses in 36K, where PPO reached 617.00 ± 16.62 Abs min^−1^ g^−1^ at 24 h after Xap (120-x). SOD, PAL, proline, and total phenolic responses also showed genotype- and time-dependent variation, with 36K generally exhibiting earlier or more pronounced defence-related responses than cv. Aras 98. The findings highlight that enhanced activation of biochemical defence mechanisms is closely associated with resistance to bacterial pathogens in common bean. These results further emphasize that the deployment of genetically resistant cultivars represents an effective, sustainable strategy for the management of bacterial diseases in bean production systems.

## 1. Introduction

The common bean (*Phaseolus vulgaris* L.), a member of the Leguminosae family, is among the most nutritionally significant and widely consumed grain legumes worldwide, utilized in multiple forms, including fresh vegetables, dry seeds, and canned and frozen products. It represents a fundamental dietary component, particularly in developing countries, owing to its high content of protein, essential minerals (K, Ca, Mg, P, and Fe), dietary fiber, and complex carbohydrates [1,2,3,4,5]. Beyond its nutritional value, the biochemical composition of common bean has also been associated with beneficial effects on human health, including the prevention and management of chronic diseases such as diabetes, obesity, cardiovascular disorders, and cancer [6].

According to FAO [7], the global cultivation area of common bean is approximately 56,208,663 hectares, with a total production of 35,345,967 tonnes. Major producing countries include China, Myanmar, India, Brazil, the United States, Indonesia, Mexico, Tanzania, Uganda, and Turkey, which ranks tenth globally. In Turkey, common bean represents the third most important pulse crop after chickpea and lentil, with a cultivation area of 132,601 hectares and a production volume of 821,074 tonnes, highlighting its considerable agricultural and economic importance.

However, common bean production is significantly constrained by bacterial diseases, particularly halo blight and common bacterial blight, caused by Psp and Xap, respectively. These pathogens have been reported to cause severe yield and quality losses across various regions of Turkey [8,9,10,11,12,13]. Under favorable environmental conditions, particularly high temperature and humidity, these bacterial diseases may lead to epidemic outbreaks, resulting in yield losses of up to 45%, depending on environmental conditions and cultivar susceptibility [14].

A key feature of plant responses to biotic stress is the rapid accumulation of reactive oxygen species (ROS), which function as both antimicrobial agents and signaling molecules [15,16]. Among defence-related enzymes, Polyphenol Oxidase (PPO) is induced upon wounding, pathogen invasion, or herbivore attack and contributes to plant defence by catalyzing the oxidation of phenolic compounds, thereby promoting lignin formation and reinforcing structural barriers against pathogens [17,18]. Antioxidant enzymes play a crucial role in maintaining ROS homeostasis; Superoxide Dismutase (SOD) catalyzes the conversion of superoxide radicals into hydrogen peroxide (H_2_O_2_) and oxygen, while catalase (CAT) detoxifies H_2_O_2_ into water and oxygen, reducing its cytotoxic effects. Similarly, ascorbate peroxidase (APX) is a key component of the antioxidant defence system, catalyzing the conversion of H_2_O_2_ into water and monodehydroascorbate [19].

Peroxidases (POXs) further contribute to plant defence through their roles as oxidoreductases, catalyzing reactions between hydrogen-donating substrates and H_2_O_2_, and are involved in critical physiological processes such as lignin biosynthesis, cell wall strengthening, hormonal regulation (particularly auxin degradation), and the modulation of indole-3-acetic acid levels during plant growth and development [20,21,22,23]. In addition, Phenylalanine Ammonia-Lyase (PAL), a key enzyme in the phenylpropanoid pathway, catalyzes the deamination of L-phenylalanine into trans-cinnamic acid and plays a central role in the biosynthesis of phytoalexins, phenolic compounds, and lignin, all of which are essential components of plant defence [24,25].

Phenolic compounds, as major secondary metabolites, are indispensable for plant growth, reproduction, and defence against pathogens. Their accumulation is widely recognized as a crucial defence response, contributing to lignin biosynthesis, reinforcement of cell walls, and the formation of physical and biochemical barriers against pathogen invasion [26,27,28].

However, limited information is available on the temporal coordination of enzymatic and phenolic defence responses in resistant and susceptible common bean genotypes challenged with both halo blight and common bacterial blight pathogens. The novelty of this study lies in the integrated evaluation of these genotype-dependent biochemical responses together with bacterial colonization and biofilm development.

In the present study, we investigated the biochemical and physiological basis of resistance in resistant and susceptible common bean genotypes exposed to bacterial infection. Specifically, we aimed to elucidate the temporal dynamics of key defence-related parameters, including ROS-associated enzymes such as SOD, PPO, and PAL, as well as total phenolic content and proline accumulation. Furthermore, plant responses were evaluated in relation to bacterial colonization and motility-associated behaviors, providing insights into the mechanistic basis of host sensitivity and resistance against these economically important pathogens.

## 2. Materials and Methods

This study was conducted between 2018 and 2023 at the laboratory of the Faculty of Agriculture at Selcuk University and the Faculty of Agriculture and Research Laboratory Application and Research Center at Igdır University. Pathogenicity and symptom development experiments, SEM analyses, and SOD assays were conducted at Selcuk University, whereas PPO, PAL, proline, total phenolic content, and bacterial motility analyses were performed at Iğdır University. The majority of the work was based on a doctoral dissertation, and additional experiments were subsequently performed to complete and strengthen the overall scope and integrity of the study.

### 2.1. Plant Material and Bacterial Strains

Seeds of common bean genotypes, including 36K (resistant genotype) and Aras 98 (susceptible cultivar), were obtained from Prof. Dr. Erdal ELKOCA, Department of Field Crops, Faculty of Agriculture, Atatürk University, and the Eastern Anatolia Agricultural Research Institute in Erzurum. This cultivar and genotype are known to differ in their responses to bacterial pathogens, with 36K exhibiting enhanced resistance and Aras 98 characterized by high susceptibility under field and controlled conditions (Table 1).

A total of four bacterial strains were used in this study, comprising two Psp (strains 510-p and 522-p) and two Xap (strains 120-x and 145-x) strains. All strains were kindly provided by Doç. Dr. Mesude Figen DÖNMEZ (Department of Plant Protection, Faculty of Agriculture, Igdır University).

Plants were cultivated under controlled growth chamber conditions with 65–70% relative humidity, a 16 h light/8 h dark photoperiod, and day/night temperatures of 23–25 °C and 15–18 °C, respectively, following established protocols [29].

### 2.2. Bacterial Inoculation and Re-Isolation of the Pathogens

Bacterial strains were cultured on Nutrient Agar (NA) medium, and suspensions were prepared in sterile distilled water to a final concentration of 10^8^ CFU mL^−1^ (OD_660_ = 0.15; Biophotometer, Eppendorf).

Inoculation procedures were conducted using distinct delivery methods tailored to each pathogen. Both Psp (510-p and 522-p) and Xap (120-x and 145-x) strains were applied to the leaf surface by spraying with a pressurized hand sprayer [29,30].

Following inoculation, Psp-treated plants were maintained at 23–25 °C, while Xap-inoculated plants were incubated at 27–28 °C in controlled growth chambers. Control plants were mock-inoculated with sterile distilled water [29].

Pathogen re-isolation was conducted from symptomatic leaf tissues to fulfill Koch’s postulates. Following symptom development, bacteria were re-isolated from symptomatic leaf tissues and identified using the same diagnostic characteristics applied to the original isolates. Recovery of bacteria consistent with the inoculated pathogen was used to complete the pathogenicity confirmation. Recovered bacterial strains were identified using a combination of biochemical, morphological, physiological (Gram reaction test, growth on YDC medium, catalase test, oxidase test, Hypersensitive Response (HR)), polymerase chain reaction (PCR) (using primers 4F/7R for Psp and RST21/RST22 for Xap), and fatty acid methyl ester (FAME) analyses [29].

### 2.3. Determination of Virulence Levels of the Bacterial Strains

The virulence of Psp and Xap strains was evaluated using the susceptible bean cultivar Aras 98. Bacterial suspensions were adjusted to a concentration of 10^8^ CFU mL^−1^ and inoculated onto plants. For each genotype/cultivar, three seeds were grown in three separate pots. The pathogenicity test was performed with three replicates and repeated three times under the same conditions. Disease progression was monitored over a period of 7–28 days post-inoculation, and symptom severity was assessed using a standardized 1–5 disease rating scale (1 = no visible symptoms; 2 = limited necrotic lesions; 3 = coalescing lesions; 4 = complete leaf necrosis; 5 = plant death). Percentage disease severity was calculated using the following formula: Disease severity (%) = [(mean disease rating − 1)/(maximum disease rating-1)] × 100, where the maximum disease rating was 5 [29,31].

### 2.4. Enzyme Activity and Total Phenolic Content Analysis Following Pathogen Infection

#### 2.4.1. Sample Collection

Leaf samples (1 g) were collected from Aras 98, 36K, and control plants at 0, 12, 24, 36, and 72 h post-inoculation (hpi). Samples were immediately frozen in liquid nitrogen and stored at −80 °C until further analysis [32].

#### 2.4.2. Polyphenol Oxidase (PPO) Activity

PPO (EC 1.14.18.1) activity was determined according to Jebara et al. [33] and Espín et al. [34]. Briefly, 1 g of fresh leaf tissue was ground in liquid nitrogen and homogenized with 6 mL of cold 50 mM sodium acetate buffer (pH 5.0). The homogenate was centrifuged at 10,000 rpm for 20 min at 4 °C, and the resulting supernatant was used as the enzyme extract. The reaction mixture contained 2 mL of 50 mM sodium acetate buffer (pH 5.0) and 100 µL of enzyme extract. The reaction was initiated by adding 100 µL of 100 mM 4-methylcatechol, and the increase in absorbance at 596 nm was recorded for 1 min. One unit of PPO activity was defined as the amount of enzyme producing an increase of 0.001 absorbance units per minute, and activity was expressed on a fresh-weight basis.

#### 2.4.3. Superoxide Dismutase (SOD) Activity

SOD (EC 1.15.1.1) activity was determined according to Sun et al. [35]. One gram of fresh leaf tissue was ground in liquid nitrogen and homogenized with 5 mL of cold 50 mM potassium phosphate buffer (pH 7.0). The homogenate was centrifuged at 10,000 rpm for 30 min at 4 °C, and the supernatant was used as the enzyme extract. The reaction system contained xanthine (0.3 mM), EDTA (0.06 mM), nitroblue tetrazolium (NBT; 150 µg L^−1^), Na_2_CO_3_ (400 mM), bovine serum albumin (1 g L^−1^), xanthine oxidase (0.166 U mL^−1^), and enzyme extract. For each assay, 2850 µL of reaction mixture, 50 µL of enzyme extract, 50 µL of distilled water, and 50 µL of xanthine oxidase were incubated for 25 min at 25 °C. The reaction was terminated by adding 50 µL of 0.8 mM CuCl_2_·2H_2_O, and absorbance was measured at 560 nm. One unit of SOD activity was defined as the amount of enzyme producing 50% inhibition of NBT reduction.

Inhibition (%)=(Blind A_560_ − Sample A_560_) / Blind A_560_ × 100

SOD Activity (U/mL)=% Inhibition / (50 × 0.05)

#### 2.4.4. Phenylalanine Ammonia-Lyase (PAL) Activity

PAL (EC 4.3.1.5) activity was determined according to McCallum and Walker [36]. One gram of fresh leaf tissue was ground in liquid nitrogen and homogenized with 5 mL of 100 mM borate buffer (pH 8.8) containing 2 mM EDTA, 5 mM β-mercaptoethanol, and 4% polyvinylpyrrolidone (PVP). The homogenate was centrifuged at 10,000 rpm for 20 min at 4 °C, and the resulting supernatant was used as the enzyme extract. The reaction mixture consisted of 3 mL of 50 mM borate buffer (pH 8.8), 0.5 mL of 20 mM L-phenylalanine, and 500 µL of enzyme extract. After incubation at 37 °C for 1 h, the reaction was terminated with 50 µL of 5 N HCl. PAL activity was determined spectrophotometrically at 290 nm using a trans-cinnamic acid calibration curve (0–100 µmol mL^−1^) and was expressed as µmol trans-cinnamic acid g^−1^ h^−1^.

#### 2.4.5. Proline Accumulation

Proline content was determined using the acid-ninhydrin method described by Bates et al. [37]. Fresh leaf tissue (0.5 g) was homogenized in 3% sulfosalicylic acid and filtered through Whatman No. 42 filter paper. Two milliliters of the filtrate was mixed with 2 mL of acid-ninhydrin reagent and 2 mL of glacial acetic acid. The acid-ninhydrin reagent was prepared by dissolving 1.25 g ninhydrin in 20 mL of 6 M phosphoric acid and 30 mL of glacial acetic acid. The reaction mixture was incubated at 100 °C for 1 h and subsequently cooled in an ice bath for 10 min. Four milliliters of toluene was then added, and the absorbance of the toluene phase was measured at 520 nm. Proline concentration was calculated using a standard calibration curve and expressed as µmol proline g^−1^ fresh weight.

$$\mu \mathrm{mol}\;\mathrm{Proline}/g\;\mathrm{fresh}\;\mathrm{weight}=\frac{\left[{(\mu g\;\mathrm{proline}\;\mathrm{ml}}^{-1}\times \;\mathrm{toluene}\;\mathrm{volume}\;\left(\mathrm{mL}\right)\;\left(115{.5\;\mu g\;\mu \mathrm{mol}}^{-1}\right)\right]}{\left(g\;\mathrm{sample}/5\right)}$$

#### 2.4.6. Determination of Total Phenolic Compounds

Total phenolic content was determined using a modified Folin–Ciocalteu method according to Singleton et al. [38]. The reaction mixture contained 6 mL distilled water; 100 µL of blank, sample, or standard solution; 500 µL Folin–Ciocalteu reagent; and 1.5 mL of 20% sodium carbonate (Na_2_CO_3_). Additionally, the final volume was adjusted to 10 mL with distilled water. After addition of the Folin–Ciocalteu reagent, the mixture was allowed to stand for 5 min before addition of sodium carbonate. The reaction mixture was then incubated for 2 h at room temperature in the dark, and absorbance was measured at 760 nm. Gallic acid was used to construct the calibration curve, and the results were expressed as gallic acid equivalents. The calibration curve showed excellent linearity (R^2^ = 0.9999). Because the Folin–Ciocalteu reagent may also react with other reducing substances, the results were interpreted as Folin–Ciocalteu-reactive reducing capacity expressed as gallic acid equivalents rather than as a specific measurement of individual phenolic compounds.

The concentration was calculated using the calibration equation: X = (Y − 0.0004)/0.0014, where Y represents absorbance at 760 nm.

### 2.5. Bacterial Colonization and Motility Following Pathogen Infection

#### 2.5.1. Scanning Electron Microscopy (SEM) Analysis of Bacterial Colonization

Bean plants (genotype 36K and cv. Aras 98) were cultivated in 15 cm diameter pots containing a sterilized mixture of peat, soil, and sand (1:1:1) under controlled greenhouse conditions (25–28 °C, 80% relative humidity, and a 10 h light/14 h dark photoperiod). At the 3–5 leaf stage, plants of cv. Aras 98 and genotype 36K were inoculated separately with either a Psp suspension containing strains 510-p and 522-p or an Xap suspension containing strains 120-x and 145-x, each adjusted to 10^8^ CFU mL^−1^, by spraying both leaf surfaces. Psp and Xap were not combined in the same inoculum. At 24 h post-inoculation, leaf samples from each genotype–pathogen combination were immediately transferred to the Advanced Technology Research and Application Center (ILTEK), located on the same university campus, for scanning electron microscopy (SEM) examination. The SEM assessment was qualitative and was used to visualize bacterial surface association and aggregation [39].

#### 2.5.2. Qualitative Assessment of Bacterial Motility

Swimming, swarming, and twitching motilities of the bacterial strains were qualitatively assessed on specifically formulated semi-solid media following previously described protocols [40,41]. The swimming medium consisted of tryptone (10 g L^−1^), yeast extract (5 g L^−1^), and agar (3 g L^−1^); the swarming medium contained nutrient broth (8 g L^−1^), glucose (5 g L^−1^), and agar (5 g L^−1^); and the twitching medium was composed of tryptone (10 g L^−1^), yeast extract (5 g L^−1^), NaCl (5 g L^−1^), and agar (10 g L^−1^), each prepared in 1000 mL distilled water. All media were adjusted to pH 7.2, sterilized by autoclaving at 121 °C for 15 min, and poured into sterile Petri dishes under aseptic conditions.

Swimming and swarming motilities were recorded as positive when characteristic radial bacterial growth from the inoculation point was observed. Twitching motility was recorded as positive based on the formation of a distinct interstitial growth zone at the agar–Petri dish interface [42,43,44]. Each assay was performed in three independent replicates, and the results were recorded qualitatively as positive or negative. No quantitative measurements of motility were performed.

### 2.6. Statistical Analysis

Each treatment consisted of three biological replicates, represented by three independently grown pots. Three independent biological replicates were used at each sampling time point, with different pots sampled at each time point. Quantitative data are expressed as mean ± standard error (SE). The data were subjected to a three-way factorial analysis of variance (ANOVA), with genotype/cultivar, bacterial strain, and sampling time considered as the main factors. The main effects and their two-way and three-way interactions were evaluated at a significance level of *p* < 0.05. When significant differences were detected, mean comparisons were performed using the least significant difference (LSD) test at the 5% significance level. Because destructive sampling was performed, different independent biological replicates were used at each sampling time point. Therefore, temporal changes were interpreted within each genotype/cultivar × bacterial strain combination, using the corresponding 0 h value as the baseline reference for that experimental group. Direct statistical comparisons among bacterial strains at the same sampling time were not considered in the revised interpretation. In the tables, different uppercase letters indicate significant differences among sampling times within each genotype/cultivar and bacterial strain. All statistical analyses were performed using IBM SPSS Statistics, version 23.

## 3. Results

In the present study, host resistance responses to Psp and Xap, two major causal agents of severe quantitative and qualitative yield losses in common bean, were comprehensively investigated at the biochemical level. Time-resolved alterations in ROS-associated antioxidant enzymes, phenylpropanoid pathway activity, proline accumulation, and total phenolic content were evaluated in both a susceptible cultivar (cv. Aras 98) and a resistant genotype (36K). In parallel, pathogen colonization dynamics in leaf tissues were considered to contextualize host defence activation.

### 3.1. Disease Severity of the Bacterial Strains

**Figure 1:**
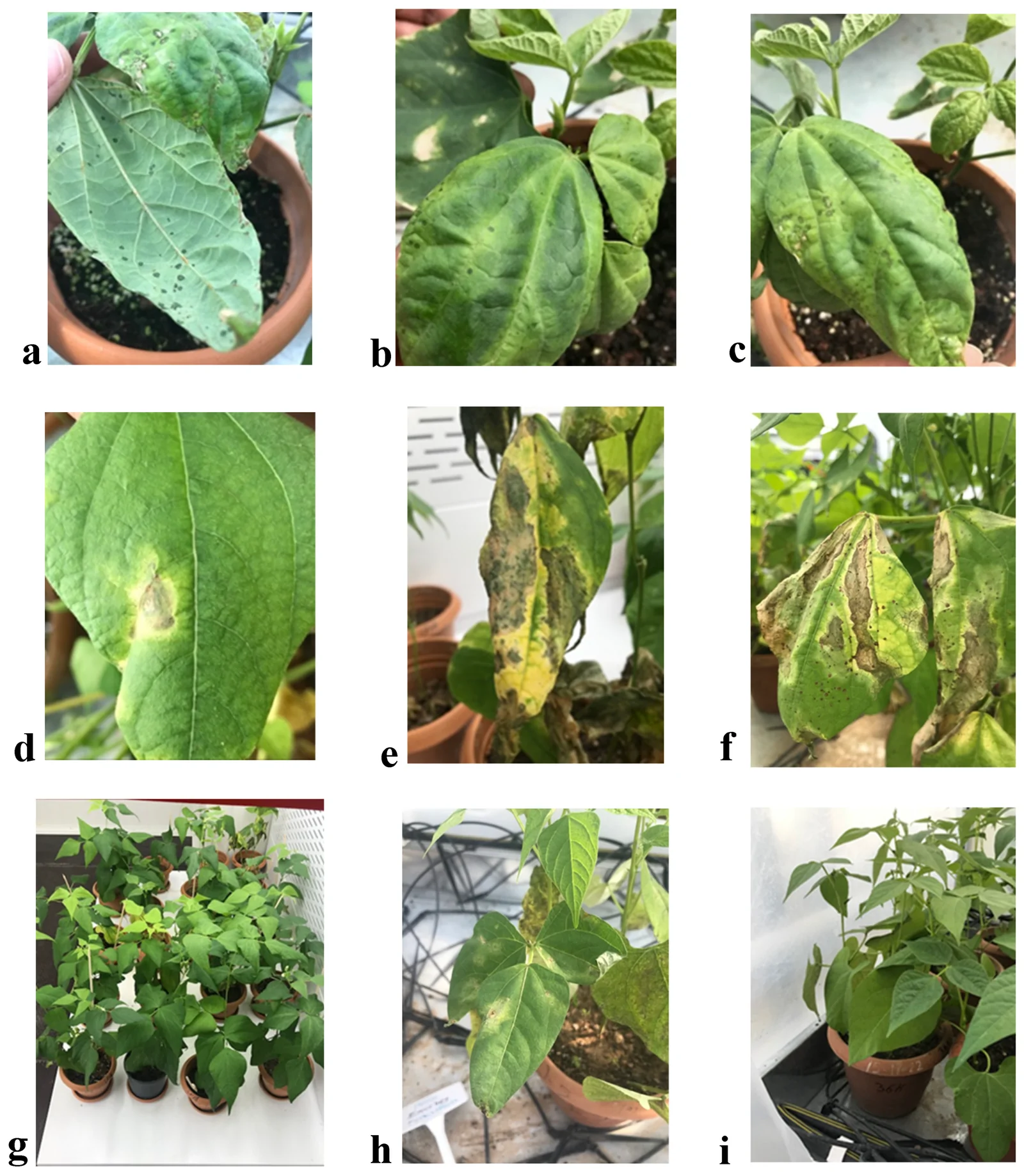
Disease symptom of Psp and Xap. (**a**,**b**) Disease symptom of Psp in Aras 98 cultivar after 7 d. (**c**) Disease symptom of Psp in Aras-98 cultivar after 28 d. (**d**) Disease symptom of *X*. *a*. pv. *phaseoli* in Aras 98 cultivar after 7 d. (**e**,**f**) Disease symptom of Xap in Aras 98 cultivar after 28 d. (**g**) Control plants of Aras 98 without bacterial inoculation. (**h**) Disease symptom of Xap in 36K genotype after 28 d. (**i**) Control plants of 36K without bacterial inoculation.

All tested strains exhibited high virulence levels, with disease severity values of 82% and 84% for Psp strains (510-p and 522-p, respectively), and 88% and 86% for Xap strains (120-x and 145-x, respectively). The disease symptoms observed in Aras 98 and 36K following inoculation with Psp and Xap are shown in Figure 1. Typical symptoms developed in Aras 98 within 7 days after inoculation with both pathogens. In contrast, no visible symptoms were observed in 36K at 7 days following either Psp or Xap inoculation. Following Xap inoculation, symptoms in 36K began to appear at 28 days, indicating delayed symptom development compared with Aras 98.

### 3.2. Polyphenol Oxidase (PPO) Activity

Because some variation was observed among the 0 h baseline values, these measurements were considered pre-infection reference values and were not interpreted as pathogen-specific responses. Accordingly, emphasis was placed on temporal changes within each treatment rather than on direct comparisons of absolute baseline values among strains. A pronounced and rapid induction of PPO activity was observed in genotype 36K following both Psp and Xap inoculations. PPO activity significantly increased as early as 12 h post-inoculation, reaching a maximum at 24 h, followed by a gradual decline at later time points (36 h and 72 h), yet remaining above control levels. In contrast, cv. Aras 98 exhibited only a moderate and transient increase, with a statistically relevant elevation primarily at 24 h, and negligible variation at most other time points, except for a strain-specific response to Psp (510-p). These findings indicate a markedly more efficient and rapidly activated oxidative defence system in the resistant genotype, suggesting PPO as a key early defence-associated marker contributing to restriction of pathogen progression.

### 3.3. Superoxide Dismutase (SOD) Activity

SOD activity displayed strong genotype- and pathogen-dependent variability. In genotype 36K, SOD activity progressively increased following Psp inoculation, reaching its highest level at 72 h, indicating sustained activation of ROS detoxification mechanisms. In response to Xap infection, however, a more limited and strain-dependent response was observed, with a significant increase only at 24 h for strain 120-x and no notable change for strain 145-x. Conversely, cv. Aras 98 exhibited a broader but less coordinated SOD activation pattern, with increases at 12, 24, and 36 h following Psp inoculation, although responses at later stages were inconsistent. Under Xap treatment, SOD activity generally increased over time, except for a transient suppression at 36 h for strain 120-x. Collectively, these results highlight a more tightly regulated and sustained oxidative stress management system in the resistant genotype compared to the susceptible cultivar.

### 3.4. Phenylalanine Ammonia-Lyase (PAL) Activity

**Figure 2:**
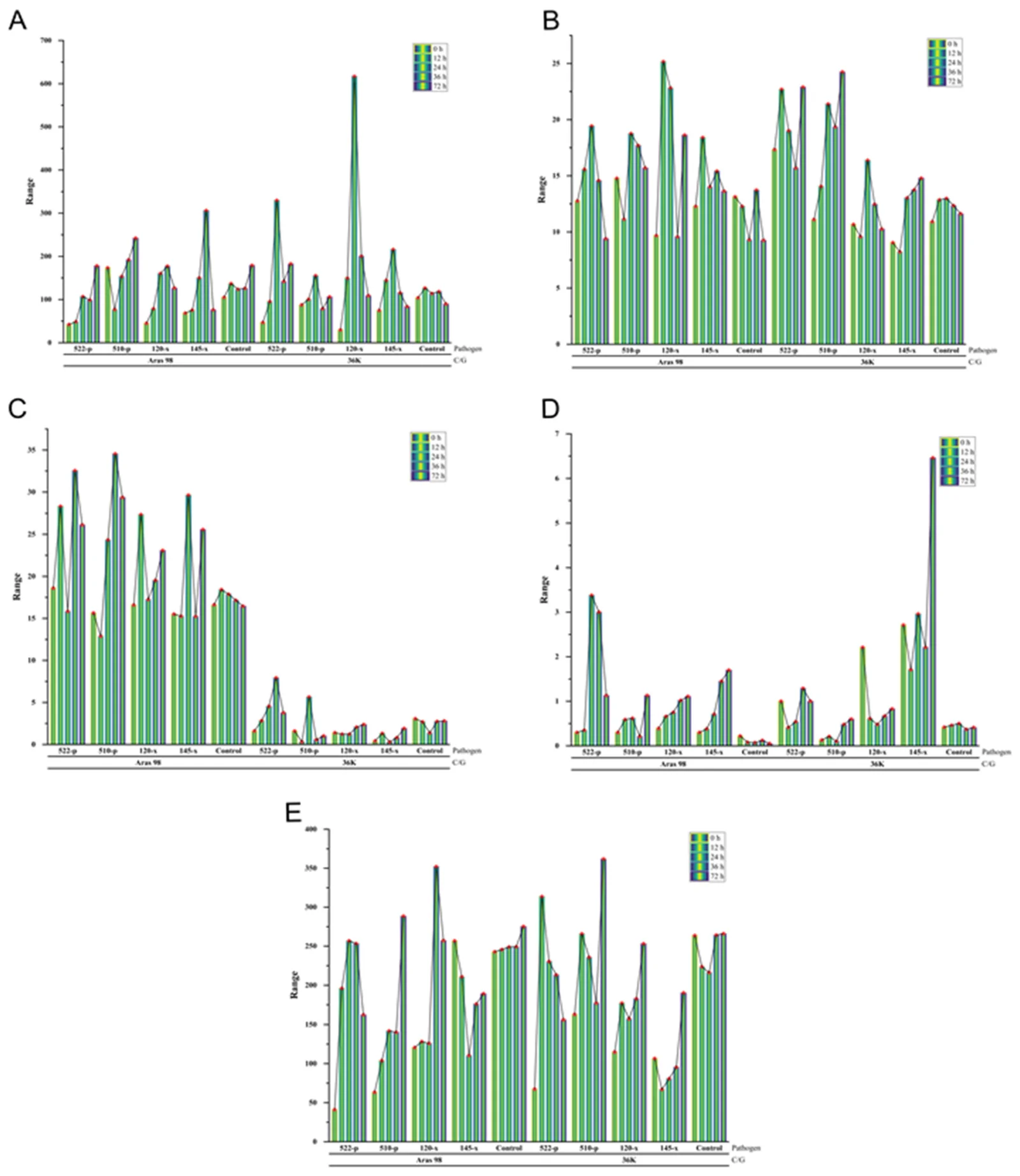
Temporal changes in defence-related enzymes and metabolites in Aras 98 and 36K cultivars following inoculation with Psp and Xap from 0 to 72 h. (**A**) PPO activity (Abs min^−1^ g^−1^), (**B**) SOD activity (U/mL), (**C**) PAL activity (μmol cinnamic acid g^−1^ h^−1^), (**D**) proline content (μmol proline g^−1^ fresh weight), and (**E**) total phenolic content (mg GAE g^−1^ fresh weight).

PAL activity exhibited a distinctly contrasting pattern between genotypes and pathogen treatments. In genotype 36K, PAL activity increased rapidly at 12 h following Psp (522-p) inoculation and continued to rise over time, whereas consistent suppression was observed across all time points following Xap infection. In cv. Aras 98, PAL activity showed a transient induction at 12 h post-Psp (522-p) inoculation, followed by a decline at subsequent time points. Under Xap treatment, PAL activity was highly variable, with a short-term increase at 12 h (120-x) and 24 h (145-x), followed by suppression at later stages, as shown in Table 2 and Figure 2. These contrasting patterns suggest a differential regulation of the phenylpropanoid pathway, potentially reflecting pathogen-specific modulation of host secondary metabolism.

### 3.5. Proline Accumulation

Proline content exhibited clear temporal and genotype-specific differences. In genotype 36K, a significant increase was detected at 36 h following Psp (522-p) inoculation, while no substantial variation was observed at other time points or under Psp (510-p) treatment. In contrast, Xap (145-x) infection triggered a sustained and progressive increase starting at 12 h and continuing throughout the experimental period. In cv. Aras 98, proline accumulation in response to Psp (522-p) began at 12 h and remained elevated at 36 h and 72 h, indicating a delayed but persistent stress response. These findings suggest that proline functions as a critical metabolic indicator of stress adaptation, particularly under sustained pathogen pressure.

### 3.6. Total Phenolic Compounds

Total phenolic content showed a robust and early induction in genotype 36K following Psp infection, with significant increases at 12 h and 24 h, and a secondary peak at 72 h for strain 510-p (Table 2 and Figure 2). However, no significant changes were observed under Xap inoculation, indicating a pathogen-specific metabolic response. In cv. Aras 98, phenolic accumulation was generally delayed and weaker, with a notable increase only at 72 h following Psp (510-p), while Xap infection resulted in a transient increase at 36 h (120-x) followed by suppression at other time points. These results strongly indicate that phenolic metabolism is more rapidly and effectively mobilized in the resistant genotype.

### 3.7. Integrated Interpretation

Overall, the resistant genotype 36K consistently demonstrated a faster, stronger, and more coordinated activation of antioxidant defence enzymes and secondary metabolite pathways compared to the susceptible cultivar. The temporal synchronization of PPO, SOD, PAL, proline, and phenolic responses suggests a highly efficient defence network that likely contributes to restricted pathogen colonization and reduced disease progression.

In contrast, the susceptible cv. Aras 98 exhibited delayed, inconsistent, and pathogen-strain-dependent responses, reflecting a less effective defence architecture. These biochemical patterns collectively underline the critical role of genotype-dependent regulation of oxidative stress management and phenylpropanoid metabolism in determining resistance outcomes against bacterial pathogens in common bean.

### 3.8. Detection of Bacterial Biofilm Colonization in Bean Cultivars Following Infection

**Figure 3:**
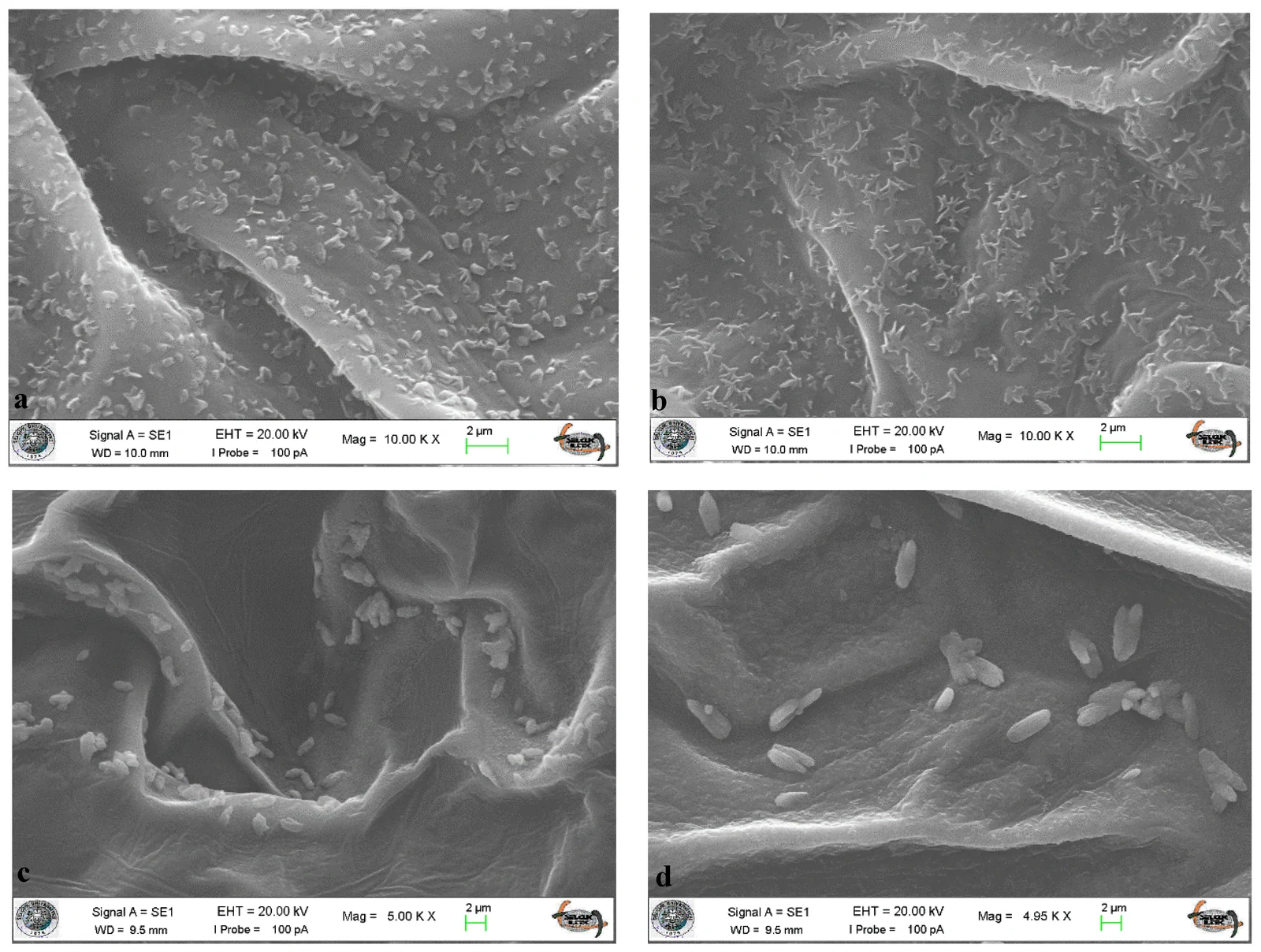
Scanning electron micrographs (SEMs) after pathogen inoculation. (**a**) SEM of Psp (510p + 522p) after 24 h in the Aras 98 cultivar. (**b**) SEM of Xap (120x + 145x) after 24 h in the Aras 98 cultivar. (**c**) SEM of Psp (510p + 522p) after 24 h in genotype 36K. (**d**) SEM of Xap (120x + 145x) after 24 h in genotype 36K.

Leaf tissues collected from the susceptible cultivar cv. Aras 98 and the resistant genotype 36K at 24 h post-inoculation with mixed suspensions of Psp (strains 510-p and 522-p) and Xap (strains 120-x and 145-x) were examined using scanning electron microscopy (SEM) to observe bacterial surface association and biofilm development (Figure 3).

SEM observations revealed qualitative differences in bacterial surface association. In the susceptible cv. Aras 98, more extensive bacterial aggregation and biofilm-like structures were observed at 24 h post-inoculation, suggesting greater surface-associated bacterial accumulation of both pathogens. In contrast, the resistant genotype 36K exhibited less extensive bacterial aggregation and fewer biofilm-like structures, suggesting qualitative differences in early surface association.

These observations suggest that genotype-related differences may be associated with distinct bacterial surface interaction patterns. In 36K, the lower extent of bacterial aggregation and biofilm-like structures may be associated with differences in early host–pathogen interactions. However, because the SEM assessment was qualitative, these observations do not establish the underlying mechanism responsible for the differences between the two genotypes.

### 3.9. Bacterial Motility Phenotypes

The tested Xap and Psp strains exhibited positive swimming, swarming, and twitching motility phenotypes under the experimental conditions used. The same qualitative responses were observed across three independent replicates. These observations are reported solely as descriptive phenotypic characteristics of the tested strains and were not interpreted as evidence of differences in colonization, biofilm formation, or virulence.

### 3.10. Principal Component Analysis and Polar Heat Map

**Figure 4:**
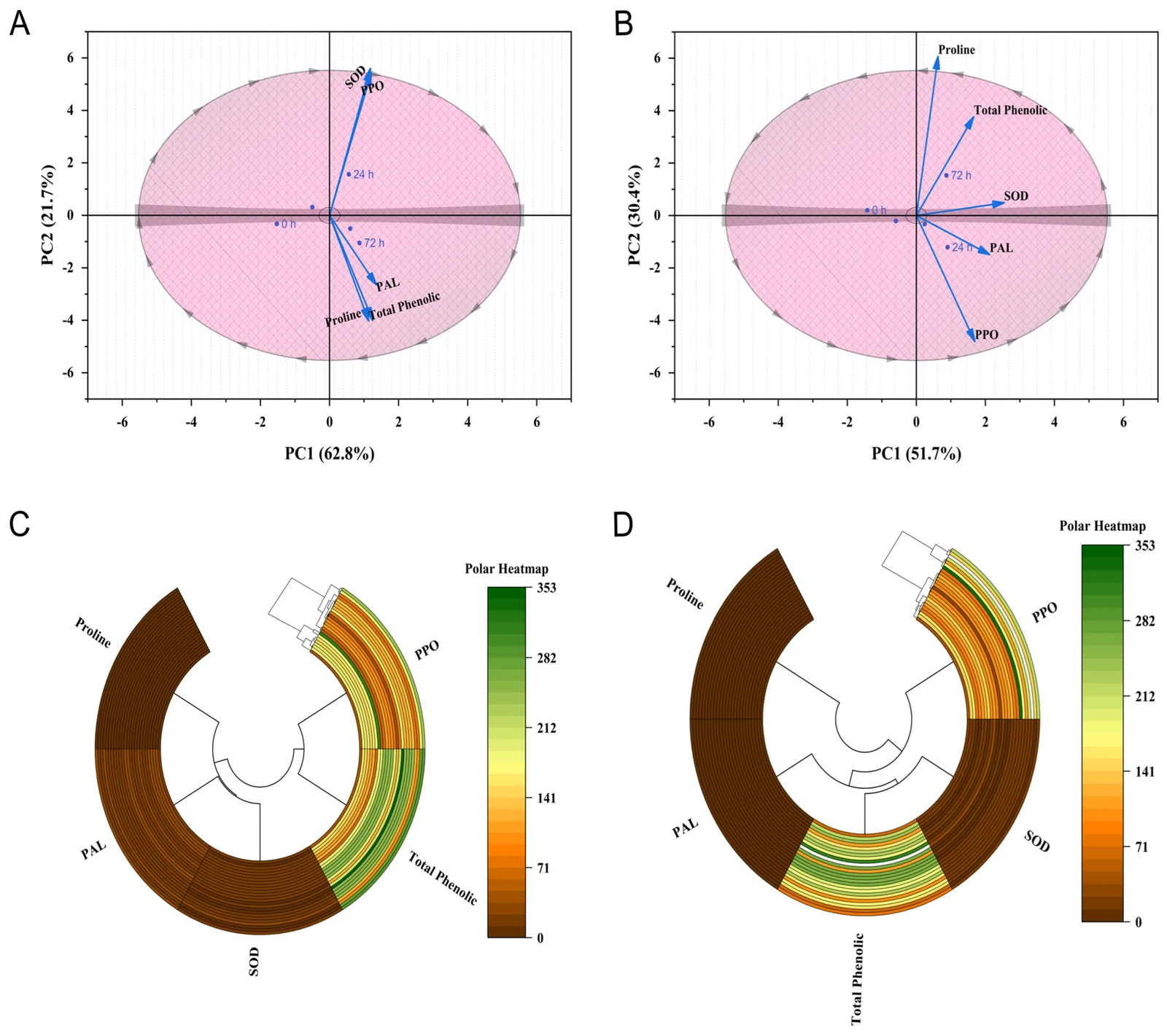
Principal component analysis (PCA) of defence-related enzymes and metabolites in Aras 98 (**A**) and 36K (**B**) cultivars, and polar heat map visualization of defence-related enzymes and metabolites in Aras 98 (**C**) and 36K (**D**) cultivars following inoculation with Xap and Psp from 0 to 72 h.

Principal component analysis (PCA) was conducted to investigate the relationships among defence-related biochemical parameters and sampling times in the Aras 98 and 36K cultivars following inoculation with Psp and Xap. In Aras 98, the first two principal components explained 84.5% of the total variation (PC1: 62.8%, PC2: 21.7%). PPO and SOD activities were strongly positively correlated, indicating coordinated antioxidant defence responses, while proline and total phenolic contents also showed a close association (Figure 4). PAL activity was positively related to proline and phenolic content but less strongly linked with PPO and SOD. Temporal separation of sampling points revealed that the 24 h stage was associated mainly with PPO and SOD activities, whereas the 72 h stage was linked to PAL activity, proline accumulation, and phenolic content, reflecting a transition from antioxidant responses to secondary metabolite production. In the 36K cultivar, the first two components explained 82.1% of the variation (PC1: 51.7%, PC2: 30.4%). Proline and total phenolics were positively correlated, while PAL and PPO activities showed coordinated responses. Early defence activation (24 h) was associated with PPO and PAL activities, whereas later responses (72 h) were characterized by increased proline and phenolic accumulation, indicating a sequential defence strategy (Figure 4).

The polar heat map revealed clear temporal changes in defence-related biochemical responses in both cultivars following pathogen inoculation. In Aras 98, total phenolic content and PPO activity displayed the greatest variability throughout the 0–72 h period, suggesting strong activation of phenolic and oxidative defence mechanisms. In contrast, SOD, PAL, and proline exhibited relatively smaller fluctuations. Similarly, in the 36K cultivar, total phenolic content and PPO activity were the most dynamic parameters, with phenolic accumulation appearing more pronounced than in Aras 98. SOD, PAL, and proline remained comparatively stable over time. Overall, the results indicate that both cultivars primarily relied on enhanced phenolic metabolism and PPO-mediated oxidative defences, although the accumulation of phenolic compounds was more evident in the 36K cultivar.

## 4. Discussion

Excessive production of ROS following pathogen attack plays a central role in plant defence signaling; however, uncontrolled ROS accumulation can lead to oxidative damage, thereby necessitating a tightly regulated antioxidant network composed of both enzymatic and non-enzymatic components. The dynamic balance between ROS generation and scavenging is therefore a critical determinant of successful defence responses in plant–pathogen interactions [28,45,46]. In this context, elucidating genotype-dependent biochemical responses provides essential insight for the development of durable disease resistance strategies.

Among defence-related enzymes, PPO has been widely associated with pathogen resistance due to its ability to catalyze the oxidation of phenolic compounds into highly reactive quinones with antimicrobial properties. The stronger PPO observed in genotype 36K is consistent with previous reports describing early PPO activation as an important component of effective plant defence [47,48,49,50]. Similar rapid PPO-mediated defence activation has been reported in tomato and mung bean systems challenged with bacterial pathogens, supporting its conserved role in plant immunity [51,52,53,54].

SOD, as the first line of enzymatic antioxidant defence, plays a pivotal role in detoxifying superoxide radicals by converting them into hydrogen peroxide and molecular oxygen [55]. Although increased SOD activity was observed in both genotypes, particularly under pathogen stress, the magnitude and temporal stability of this response varied significantly. This supports previous findings indicating that elevated ROS production can occur in both resistant and susceptible interactions; however, the effectiveness of downstream detoxification systems determines disease outcome rather than ROS production alone [56,57,58]. While some studies report higher SOD activity in resistant genotypes [59], others indicate stronger responses in susceptible cultivars, reflecting the complexity of ROS regulation in plant immunity [60,61,62]. The present findings support the notion that SOD activity alone is not a definitive indicator of resistance, but rather part of a broader, coordinated antioxidant network.

PAL, a key regulatory enzyme of the phenylpropanoid pathway, is widely recognized for its role in the biosynthesis of phenolics, lignin precursors, and phytoalexins that contribute to structural and chemical defence. The observed induction of PAL activity, particularly in genotype 36K following Psp infection, is consistent with its established role in enhancing disease resistance through reinforcement of cell walls and accumulation of antimicrobial metabolites [63,64,65]. This delayed peak compared to previously reported 24 h maxima [66] may reflect pathogen-specific modulation of host metabolism and differential infection dynamics. Similar genotype-dependent variations in PAL activity have been reported in tomato and bean systems under bacterial challenge [53,67,68,69].

Proline accumulation, a well-established marker of abiotic and biotic stress responses, exhibited clear genotype- and pathogen-dependent patterns. The increased proline levels observed in both genotypes under infection conditions are consistent with its multifunctional role in osmotic adjustment, redox buffering, and stress signaling [70,71,72,73]. The association between proline metabolism and protein turnover further supports the hypothesis that pathogen-induced metabolic reprogramming contributes to amino acid accumulation during defence responses [74].

Phenolic compounds represent one of the most functionally diverse groups of secondary metabolites involved in plant defence. Their rapid accumulation at infection sites is a key component of early defence responses, contributing to pathogen restriction through antimicrobial activity and reinforcement of structural barriers [75,76,77,78]. These findings are consistent with previous studies demonstrating genotype-dependent timing of phenolic accumulation in plant–bacterial interactions [79].

A particularly important aspect of this study is the complementary evaluation of biochemical responses together with qualitative SEM observations of bacterial surface association. At 24 h post-inoculation, more extensive bacterial aggregation and biofilm-like structures were observed on cv. Aras 98, whereas genotype 36K exhibited less extensive aggregation and fewer biofilm-like structures. These observations indicate qualitative differences in early bacterial surface association between the two genotypes These qualitative observations are consistent with previous reports indicating higher bacterial colonization in susceptible bean cultivar [80]. Biofilm formation is a well-documented bacterial strategy associated with surface attachment, survival, and host colonization. However, because bacterial cell numbers, aggregate density, surface coverage, and extracellular matrix production were not quantitatively measured in the present study, the SEM observations should not be interpreted as direct evidence of reduced colonization or suppressed biofilm formation in 36K. Rather, they provide complementary qualitative information on differences in host–pathogen surface interactions under the conditions examined.

In contrast, the susceptible cv. Aras 98 exhibits delayed and less coordinated responses, which likely facilitate successful infection and disease progression.

From an applied perspective, these findings highlight the critical importance of genetic resistance in sustainable plant disease management. The identification of genotype-specific biochemical signatures, particularly early activation of PPO, SOD, PAL, phenolics, and proline metabolism, provides valuable physiological markers for resistance screening. Moreover, the 36K genotype represents a promising genetic resource for breeding programs aimed at improving resistance against bacterial pathogens in common bean.

Ultimately, a deeper understanding of the molecular and biochemical basis of plant–pathogen interactions will be essential for developing durable, environmentally sustainable disease control strategies. Targeting ROS homeostasis and secondary metabolite pathways offers a particularly promising direction for future resistance breeding efforts in legume crops.

The use of chemical control strategies against bacterial pathogens is increasingly restricted due to rising production costs, environmental concerns, and potential adverse effects on non-target organisms. Consequently, the development and deployment of disease-resistant cultivars represent the most effective, economical, and environmentally sustainable approach for long-term disease management. In this context, elucidating plant defence mechanisms at the molecular level is critical for guiding breeding strategies aimed at improving both agronomic performance and disease resistance.

## 5. Conclusions

This study revealed genotype-dependent differences in the temporal responses of SOD, PPO, PAL, proline, and total phenolic compounds following separate inoculations with Psp and Xap. The combined evaluation of biochemical responses and qualitative SEM observations revealed genotype-associated differences in biochemical defence responses and early bacterial surface interactions. The magnitude and timing of these biochemical responses differed between genotype 36K and cv. Aras 98 and varied according to pathogen and sampling time. Qualitative SEM observations revealed differences in bacterial surface association and aggregation between the two genotypes. Importantly, genotype 36K represents promising material for further evaluation and validation in studies of bacterial disease resistance in common bean. The observed changes in SOD, PPO, PAL, phenolic compounds, and proline may represent candidate biochemical traits for further validation across broader genotype panels and independent populations.

## Figures and Tables

**Table 1:** Seed characteristics of bean cultivars/genotypes used in study.

Genotype/Cultivar	1000-Seed Weight (g)	Seed Shape	Seed Color	Plant Growth Habit
36-K	245.2	Elliptic	Dark red	Semi-climbing
Aras-98	454.0	Oblong	White	Bush

**Table 2:** PPO, SOD and PAL enzymes, and proline and total phenolic compounds in bean genotype/cultivar at 0, 12, 24, 36 and 72 h after Psp and Xap inoculation.

Enzyme	C/G	Pathogen	Hours After Pathogen Inoculation				
			0.	12.	24.	36.	72.
**Polyphenol oxidase (PPO) activity** **(Abs min^−1^ g^−1^)**	**Aras 98**	**522-p**	42.33 ± 3.92 **B**	48.66 ± 3.28 **B**	107.00 ± 3.51 **A**	98.66 ± 3.17 **AB**	178.00 ± 6.11 **A**
		**510-p**	173.66 ± 15.30 **A**	77.00 ± 11.35 **B**	153.33 ± 11.40 **A**	192.00 ± 8.18 **A**	242.33 ± 9.68 **A**
		**120-x**	45.00 ±0.57 **C**	78.66 ± 8.29 **BC**	160.33 ± 28.95 **A**	177.33 ± 8.83 **A**	126.66 ± 10.83 **ABC**
		**145-x**	68.66 ± 1.76 **B**	75.33 ± 9.52 **B**	150.33 ± 2.02 **B**	306.33 ± 191.83 **A**	76.33 ± 20.99 **B**
		**Control**	105.00 ± 0.57 **A**	137.00 ± 20.03 **A**	123.66 ± 29.80 **A**	126.00 ± 33.08 **A**	179.33 ± 4.33 **A**
	**36K**	**522-p**	47.00 ± 6.24 **D**	95.33 ± 6.35 **CD**	329.66 ± 6.88 **A**	142.00 ± 11.53 **BC**	182.66 ± 10.47 **B**
		**510-p**	87.66 ± 8.83 **A**	100.66 ± 5.45 **A**	155.33 ± 0.88 **A**	78.66 ± 6.35 **A**	106.33 ± 2.84 **A**
		**120-x**	29.66 ± 3.28 **D**	149.66 ± 9.24 **BC**	617.00 ± 16.62 **A**	200.66 ± 9.83 **B**	109.00 ± 16.37 **CD**
		**145-x**	74.66 ± 6.35 **AB**	144.66 ± 10.91 **B**	216.00 ± 16.64 **AB**	115.66 ± 4.17 **A**	83.66 ± 1.20 **AB**
		**Control**	104.00 ± 5.56 **B**	126.67 ± 6.83 **A**	114.00 ± 0.57 **A**	118.33 ± 7.35 **B**	89.66 ± 5.17 **A**
**Superoxide Dismutase** **(SOD) activity** **(** **U/mL)**	**Aras 98**	**522-p**	12.76 ± 0.81 **BC**	15.58 ± 0.93 **AB**	19.44 ± 0.52 **A**	14.58 ± 1.88 **ABC**	9.40 ± 0.00 **C**
		**510-p**	14.78 ± 1.59 **AB**	11.14 ± 0.94 **B**	18.76 ± 2.51 **A**	17.70 ± 1.75 **A**	15.70 ± 2.51 **AB**
		**120-x**	9.68 ± 1.11 **C**	25.17 ± 0.00 **A**	22.82 ± 1.56 **AB**	9.57 ± 2.83 **C**	18.63 ± 0.00 **B**
		**145-x**	12.28 ± 0.31 **B**	18.41 ± 3.84 **A**	14.01 ± 1.85 **AB**	15.42 ± 0.85 **AB**	13.63 ± 0.00 **AB**
		**Control**	13.13 ± 1.86 **A**	12.28 ± 2.21 **A**	9.30 ± 2.34 **A**	13.73 ± 0.00 **A**	9.26 ± 0.00 **A**
	**36K**	**522-p**	17.34 ± 2.82 **B**	22.70 ± 0.25 **A**	19.01 ± 4.94 **AB**	15.69 ± 2.28 **B**	22.90 ± 0.70 **A**
		**510-p**	11.11 ± 1.82 **B**	14.06 ± 1.31 **B**	21.40 ± 1.93 **A**	19.35 ± 2.85 **A**	24.25 ± 4.64 **A**
		**120-x**	10.67 ± 1.21 **B**	9.58 ± 0.76 **B**	16.38 ± 1.15 **A**	12.47 ± 0.03 **AB**	10.26 ± 1.43 **B**
		**145-x**	9.05 ± 0.37 **BC**	8.22 ± 0.00 **C**	13.03 ± 0.38 **ABC**	13.75 ± 0.00 **AB**	14.79 ± 1.93 **A**
		**Control**	10.91 ± 1.54 **A**	12.86 ± 0.38 **A**	12.98 ± 0.90 **A**	12.35 ± 1.68 **A**	11.62 ± 0.94 **A**
**Phenylalanine Ammonia Lyase (PAL) activity** **(μmol cinnamic acid** g^−1^ h**^−1^)**	**Aras 98**	**522-p**	18.60 ± 1.07 **C**	28.31 ± 0.90 **AB**	15.84 ± 0.40 **C**	32.55 ± 0.58 **A**	26.12 ± 0.00 **B**
		**510-p**	15.65 ± 1.05 **C**	12.88 ± 0.72 **C**	24.33 ± 6.70 **B**	34.54 ± 4.43 **A**	29.36 ± 1.00 **AB**
		**120-x**	16.59 ± 1.14 **C**	27.35 ± 0.00 **A**	17.24 ± 1.72 **BC**	19.53 ± 4.33 **BC**	23.08 ± 1.28 **AB**
		**145-x**	15.51 ± 3.18 **B**	15.26 ± 0.00 **B**	29.63 ± 1.19 **A**	15.23 ± 1.99 **B**	25.57 ± 1.64 **A**
		**Control**	16.62 ± 0.83 **A**	18.42 ± 1.01 **A**	17.88 ± 1.38 **A**	17.15 ± 1.58 **A**	16.46 ± 1.79 **A**
	**36K**	**522-p**	16.6290 ± 2.72 **BC**	15.1220 ± 0.43 **C**	19.0927 ± 1.53 **BC**	22.3903 ± 0.84 **B**	32.2500 ± 3.37 **A**
		**510-p**	15.6810 ± 0.15 **B**	17.9840 ± 1.15 **B**	30.1857 ± 1.34 **A**	34.9160 ± 0.41 **A**	30.5967 ± 1.15 **A**
		**120-x**	14.2127 ± 1.18 **B**	17.8910 ± 3.53 **B**	13.5150 ± 0.27 **B**	18.2850 ± 0.66 **B**	32.4073 ± 3.29 **A**
		**145-x**	24.0160 ± 0.05 **AB**	18.8940 ± 0.98 **B**	19.5083 ± 2.24 **AB**	25.7253 ± 1.88 **A**	20.5457 ± 0.00 **AB**
		**Control**	12.4917 ± 0.02 **B**	18.2113 ± 0.22 **AB**	19.1563 ± 0.82 **A**	12.9363 ± 0.88 **B**	20.3073 ± 4.49 **A**
**Proline content** **(μmol proline g^−1^ fresh weight)**	**Aras 98**	**522-p**	0.30 ± 0.01 **B**	0.35 ± 0.01 **B**	3.38 ± 0.01 **A**	3.00 ± 0.01 **A**	1.13 ± 0.01 **B**
		**510-p**	0.30 ± 0.01 **A**	0.59 ± 0.01 **A**	0.62 ± 0.00 **A**	0.21 ± 0.10 **A**	1.13 ± 0.01 **A**
		**120-x**	0.39 ± 0.00 **A**	0.66 ± 0.01 **A**	0.75 ± 0.01 **A**	1.02 ± 0.02 **A**	1.11 ± 0.01 **A**
		**145-x**	0.30 ± 0.00 **B**	0.38 ± 0.07 **B**	0.71 ± 0.13 **AB**	1.45 ± 0.37 **A**	1.70 ± 0.56 **A**
		**Control**	0.22 ± 0.09 **A**	0.08 ± 0.03 **A**	0.07 ± 0.01 **A**	0.12 ± 0.01 **A**	0.05 ± 0.01 **A**
	**36K**	**522-p**	1.00 ± 0.02 **A**	0.41 ± 0.02 **A**	0.54 ± 0.02 **A**	1.29 ± 0.02 **A**	1.00 ± 0.01 **A**
		**510-p**	0.13 ± 0.01 **A**	0.21 ± 0.02 **A**	0.10 ± 0.02 **A**	0.48 ± 0.01 **A**	0.60 ± 0.01 **A**
		**120-x**	2.21 ± 0.02 **A**	0.61 ± 0.07 **B**	0.48 ± 0.02 **B**	0.67 ± 0.02 **B**	0.83 ± 0.02 **B**
		**145-x**	2.71 ± 0.13 **B**	1.71 ± 0.39 **C**	2.96 ± 0.45 **B**	2.21 ± 0.49 **BC**	6.46 ± 2.15 **A**
		**Control**	0.42 ± 0.01 **A**	0.46 ± 0.01 **A**	0.50 ± 0.01 **A**	0.37 ± 0.01 **A**	0.41 ± 0.02 **A**
**Total Phenolic Compound** **(mg GAE g^−1^ fresh) weight)**	**Aras 98**	**522-p**	41.1 ± 0.04 **D**	196.38 ± 4.78 **B**	256.86 ± 0.41 **A**	253.31 ± 0.11 **A**	162.17 ± 0.04 **C**
		**510-p**	63.36 ± 0.04 **D**	103.81 ± 0.26 **C**	141.86 ± 0.04 **B**	140.24 ± 0.06 **B**	288.52 ± 1.26 **A**
		**120-x**	120.57 ± 0.29 **C**	128.21 ± 0.04 **C**	125.90 ± 0.07 **C**	352.1 ± 23.5 **A**	257.24 ± 0.02 **B**
		**145-x**	256.86 ± 2.58 **A**	210.67 ± 6.68 **A**	110.40 ± 2.36 **D**	175.90 ± 5.49 **C**	189.24 ± 7.81 **B**
		**Control**	242.83 ± 0.06 **A**	245.57 ± 2.47 **A**	248.93 ± 1.24 **A**	249.12 ± 2.85 **A**	275.24 ± 0.69 **A**
	**36K**	**522-p**	67.45 ± 0.40 **E**	313.64 ± 0.33 **A**	230.43 ± 3.27 **B**	213.21 ± 0.14 **C**	155.95 ± 0.06 **D**
		**510-p**	162.67 ± 0.17 **E**	265.83 ± 0.06 **B**	235.70 ± 12.90 **C**	177.29 ± 0.16 **D**	361.86 ± 0.36 **A**
		**120-x**	114.88 ± 0.08 **D**	177.10 ± 0.02 **B**	157.24 ± 0.20 **C**	182.74 ± 0.05 **B**	253.05 ± 7.87 **A**
		**145-x**	106.60 ± 0.10 **B**	66.98 ± 0.62 **D**	80.67 ± 0.42 **C**	95.45 ± 0.33 **B**	190.19 ± 6.78 **A**
		**Control**	263.60 ± 0.08 **A**	223.62 ± 0.02 **B**	216.21 ± 0.00 **B**	264.24 ± 0.02 **A**	266.00 ± 0.04 **A**

A, B, C, D: Comparison of sampling times within each genotype/cultivar and bacterial strain.

## Data Availability

All data and materials presented in this study are available from the corresponding author upon reasonable request.
